# Obtaining Highly Active Catalytic Antibodies Capable of Enzymatically Cleaving Antigens

**DOI:** 10.3390/ijms232214351

**Published:** 2022-11-18

**Authors:** Tamami Nonaka, Hiroaki Taguchi, Taizo Uda, Emi Hifumi

**Affiliations:** 1 Institute for Research Management, Oita University, 700 Dannoharu, Oita 870-1192, Japan; 2 Faculty of Pharmaceutical Sciences, Suzuka University of Medical Science, 3500-3 Minamitamagaki-cho, Suzuka 510-0293, Japan; 3 Nanotechnology Laboratory, Institute of Systems, Information Technologies and Nanotechnologies (ISIT), 4-1 Kyudai-shinmachi, Fukuoka 879-5593, Japan; 4 Research Center for GLOBAL/LOCAL Infectious Diseases, Oita University, 700 Dannoharu, Oita 870-1192, Japan

**Keywords:** catalytic antibody light chain, Ni-NTA affinity column, size-exclusion chromatography, amyloid-beta, PD-1, FRET substrate

## Abstract

A catalytic antibody has multiple functions compared with a monoclonal antibody because it possesses unique features to digest antigens enzymatically. Therefore, many catalytic antibodies, including their subunits, have been produced since 1989. The catalytic activities often depend on the preparation methods and conditions. In order to elicit the high catalytic activity of the antibodies, the most preferable methods and conditions, which can be generally applicable, must be explored. Based on this view, systematic experiments using two catalytic antibody light chains, #7TR and H34, were performed by varying the purification methods, pH, and chemical reagents. The experimental results obtained by peptidase activity tests and kinetic analysis, revealed that the light chain’s high catalytic activity was observed when it was prepared under a basic condition. These data imply that a small structural modulation of the catalytic antibody occurs during the purification process to increase the catalytic activity while the antigen recognition ability is kept constant. The presence of NaCl enhanced the catalytic activity. When the catalytic light chain was prepared with these preferable conditions, #7TR and H34 hugely enhanced the degradation ability of Amyloid-beta and PD-1 peptide, respectively.

## 1. Introduction

A catalytic antibody has multiple functions compared with a monoclonal antibody (mAb) because it possesses unique features to degrade antigen molecules enzymatically and specifically recognize them. Based on this concept, many naturally occurring catalytic antibodies, including their subunits, such as light and heavy chains, have been produced by researchers since 1989 [1,2,3,4,5]. These catalytic antibodies have interesting functions to hydrolyze targeted peptides [1,5,6,7,8], nucleotides [2,4,9], some physiologically active molecules [3,10,11,12,13], and several viral and bacterial antigenic proteins [14,15,16,17,18,19,20,21,22]. In the development of the natural catalytic antibody, several difficult problems have existed. Some of the issues have been solved in these two decades. With respect to the cleaving mechanism of catalytic antibodies, a similar mechanism as that of serine proteases can be applicable, according to studies by site-directed mutagenesis [23,24] and X-ray crystallography [25,26,27] and X-ray crystallography [25,26,27]. The structural diversity of catalytic antibodies was previously an issue, which has resulted in the production of antibody drugs, for example, trastuzumab [28,29]. This problem has been solved in recent years by using copper ions during the preparation process [30,31,32]. The most difficult and important issue is how we can easily obtain and/or produce the catalytic antibody we want, which was partly solved by mutating or deleting Pro95 residue residing at CDR-3 in the antibody light chain [33]. Although many studies have solved those problems for two decades, one more important issue, how to obtain a highly active form of the catalytic antibody, remains. The activity of the catalytic antibody is varied depending on the preparation methods and conditions, as with the preparation of enzymes. If those are not appropriate, the catalytic activity is low or lost [34]. Many researchers studying catalytic antibodies have faced this difficult phenomenon. The present study aimed to investigate and determine the most preferable methods and conditions to obtain high and/or intrinsic catalytic activity along with reproducibility.

## 2. Results

**Figure 1:**
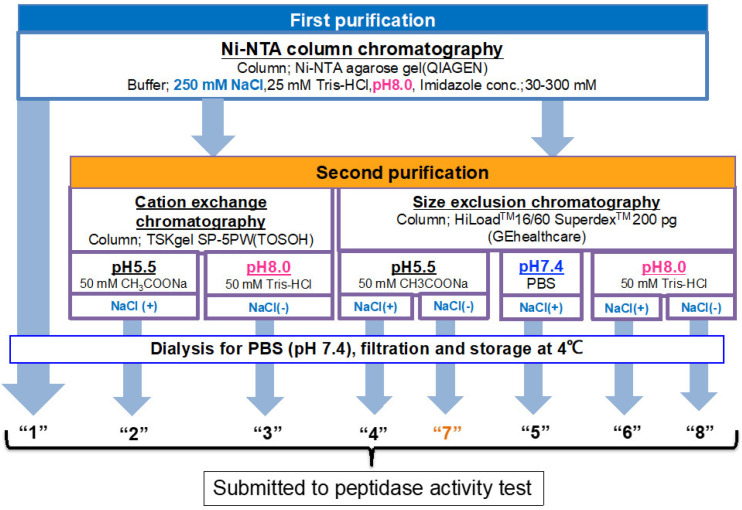
**Overall strategy and the flow chart for systematic experiments.** Ni-NTA affinity chromatography was performed for all samples as the first purification step. The sample obtained by Ni-NTA affinity chromatography is designated as route “1.” For the second purification step, a cation exchange or size exclusion column chromatography was employed, in which the sample was purified under various conditions by changing pH and with or without NaCl. All samples were dialyzed against PBS (pH 7.4) twice and filtered for sterilization. Finally, the samples were subjected to a peptidase activity test.

As presented in Figure 1, the experiments were planned to investigate or determine the optimal purification condition, and tested factors were pH and the presence of chemicals such as NaCl and Tween. Briefly stated, after recovering the supernatant via centrifugation of the cell lysate, all samples used in the present study were submitted to Ni-NTA column chromatography at a pH of 8.0 as the first purification step. For the second purification step, cation-exchange or size-exclusion chromatography was utilized, in which the sample was purified under various conditions by changing the pH and with or without NaCl and Tween 20.

### 2.1. #7TR Light Chain

#### 2.1.1. Ni-NTA Column Chromatography (Route “1”) 

**Figure 2:**
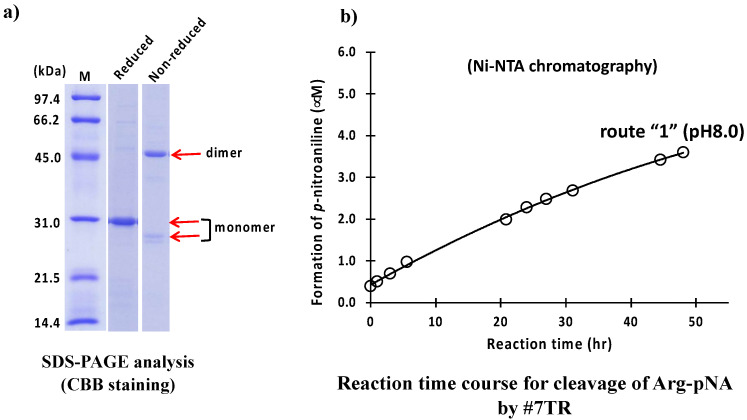
**SDS-PAGE and peptidase activity by route “1”.** (**a**) **SDS-PAGE analysis.** Bands were visualized by CBB staining. Under the reduced condition, only the ~30 kDa band corresponding to the monomer was detected as a single band. Bands other than the monomer band of the light chains were barely observed, suggesting that the #7TR light chains were highly purified. Under the non-reduced condition, bands at ~46 kDa corresponding to the dimer and at ~26 kDa corresponding to the monomer were observed. (**b**) **Peptidase activity test.** Reaction conditions; #7TR: 6.8 μM, Arg pNA: 200 μM, Reaction temperature: 37 °C, Reaction volume: 200 µL, Arg(R)-pNA (trypsin-like substrate) was used as the substrate in 50 mM/Tris-100 mM/Glycine-Tween-20 buffer (TGT) buffer. The reaction was performed in triplicate in a 96-well micro-plate.

A catalytic antibody light chain, #7TR, belonging to Subgroup II of the human antibody light chain by Kabat’s classification (the amino acid [aa] sequence: see [35]), was used as the sample. #7TR is a unique catalytic antibody in which the amino acids at Thr^29^ and Arg^30^ mutated from Gly^29^ and Tyr^30^ of #7wt (wild type), respectively. From these mutations, #7TR acquired its peptidase activity [35]. Based on these data, we employed the #7TR light chain in this study to investigate the most preferable preparation methods and conditions. The detailed purification conditions were described in the Materials and Methods. Briefly, #7TR supernatant via centrifugation of the cell lysate was recovered, and the sample was submitted to Ni-NTA column chromatography as a first step purification, equilibrated with 25 mM Tris-HCl, pH 8.0, containing 250 mM NaCl. Elution was performed by increasing the concentration of imidazole. In Figure S1, the detailed procedure, chromatography, and SDS-PAGE are presented. After the Ni-NTA column chromatography (purification route “1”) was completed, an aliquot of a solution of 50 mM CuCl_2_ was added into the eluent and allowed to react for 16 h to make a dimer form. After the solution was concentrated to 3–6 mg/mL, EDTA was added to remove the Cu(II). Then the sample was submitted to dialysis against PBS (pH 7.4) twice, followed by filtration (0.2 µm). The purity and the form of purified #7TR (by route “1”) were examined using SDS-PAGE analysis with Coomassie brilliant blue (CBB) staining (Figure 2a). Under the reduced condition, the bands observed at approximately ~30 kDa corresponded to the monomer of the #7TR antibody light chain, whereas the other bands were barely observed, indicating the high purity of #7TR (>95%). Under the non-reduced condition, the band at approximately 46 kDa and the faint band at approximately 26 kDa corresponded to the dimer and monomer of the #7TR light chain, respectively. 

The peptidase activity of the #7TR light chain (6.8 μM) was tested using a synthetic substrate, Arg-pNA (R-pNA), which is a trypsin-like substrate molecule. The reaction time course for the R-pNA cleavage is shown in Figure 2b. The #7TR gradually hydrolyzed the synthetic substrate R-pNA, resulting in the formation of *p*-nitroaniline. The degradation reaction was monitored for up to 48 h. The reaction velocity was 0.10 μM/h at 6.8 μM of #7TR. The peptidase activity was comparable to that of 22F6 human catalytic antibody (reaction velocity; 0.09 μM/h at the concentration of 4.0 μM 22F6) [20].

#### 2.1.2. Cation Exchange Chromatography (as a Second Step Purification) 

**Figure 3:**
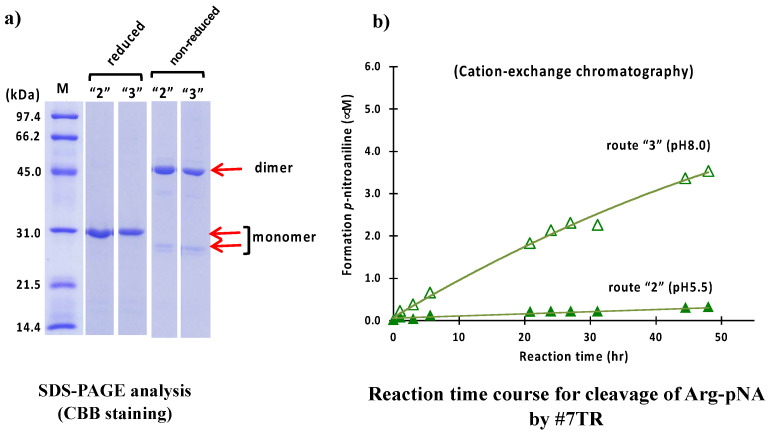
**SDS-PAGE and peptidase activity by route “2” and “3”.** (**a**) **SDS-PAGE analysis.** Bands were visualized by CBB staining. Under both reduced and non-reduced conditions, bands such as ~30 kDa, a faint ~26 kDa, and the visible~ 46 kDa observed in Figure 2a were detected. #7TR was highly purified. (**b**) **peptidase activity.** The reaction conditions were the same as those performed in Figure 2b. The catalytic activity of #7TR purified at pH 8.0 by route “3” showed moderate activity (open triangle), as observed in Figure 2b. In contrast, #7TR purified at pH 5.5 by route “2” was very low (solid triangle).

As a second purification step after the Ni-NTA column chromatography, cation exchange chromatography was carried out at pH 5.5 (route “2”) and 8.0 (route “3”). Briefly stated, in the case of pH 5.5 (route “2”), the solution, including the #7TR and copper ion, was dialyzed against a Na acetate buffer, pH 5.5, for overnight and subjected to cation exchange chromatography with a gradient of NaCl solution on the purification apparatus. In the case of pH 8.0 (route “3”), the dialysis of the solution, including the light chain and copper ion, was performed against Tris-HCl buffer (pH 8.0), and then the sample was applied to cation exchange chromatography. The results of the SDS-PAGE analysis for route “2” are shown in Figure 3a. 

In both cases of route “2” and “3” under non-reduced conditions, the band at 26 kDa, corresponding to the monomer was faint, but the band at 46 kDa, corresponding to the dimer, was robust, indicating that the dimer was dominant in the solution. Under the reduced condition, only the band at ~30 kDa. corresponding to the monomer, was observed, and no other bands were detected.

The peptidase activities of the #7TR light chain purified with routes “2” and “3” were examined in the same way as previously described. The reaction time courses are shown in Figure 3b. The catalytic activity of #7TR purified at pH 8.0 (route “3”) showed a similar profile as that observed with route “1” (Ni-NTA chromatography at pH 8.0). In contrast, the activity at pH 5.5was low. 

#### 2.1.3. Size-Exclusion Chromatography (as the Second Step Purification)

Size-exclusion chromatography was another second purification step, where the purification pH was measured at pH 5.5 (route “4”), 7.4 (route “5”), and 8.0 (route “6”), was performed. Briefly, in the preparation of pH 5.5 (route “4”), the solution, including the #7TR and copper ion, was dialyzed against a Na acetate buffer with NaCl (pH 5.5) overnight and subjected to size-exclusion chromatography on the AKTA system, with a Na acetate buffer including NaCl (pH 5.5) used as the eluent solvent. In the case of pH 7.4 (route “5”), PBS adjusted with pH 7.4 was used instead of Na acetate buffer in the dialysis buffer and the eluent solvent. In the case of pH 8.0 (route “6”), a Tris-HCl including NaCl (pH 8.0) was used for the dialysis as well as the eluent solvent. 

**Figure 4:**
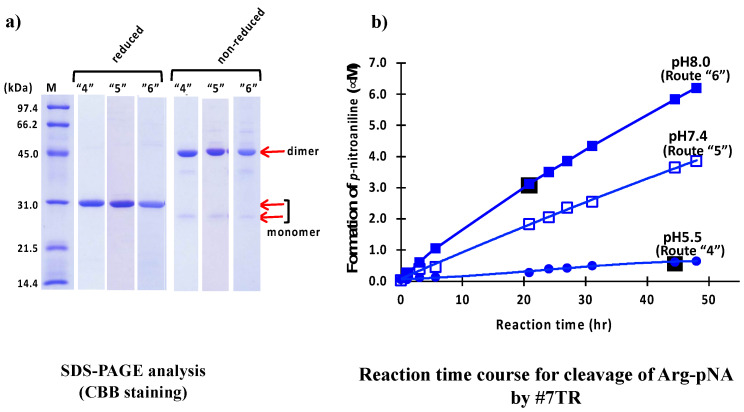
**#7TR purified by routes “4”, “5,” and “6”.** (**a**) **SDS-PAGE analysis.** Bands were visualized by CBB staining. Under both reduced and non-reduced conditions, bands such as ~30 kDa, a faint ~26 kDa, and the visible ~46 kDa as those observed in Figure 2a and Figure 3a were detected for three preparation routes. In all cases, #7TR was highly purified. (**b**) **peptidase activity.** The reaction conditions were the same as those in Figure 2b and Figure 3b. The catalytic activity of #7TR purified route “4” at pH 5.5 (solid circle), route “5” at pH 7.4 (open square), and “6 at pH 8.0 (solid square) was examined. The activity of route “6” at pH 8.0 was very high, followed by route “5” at pH 7.4 showing the moderate activity. That by route “4” at pH 5.5 was very low.

The results of SDS-PAGE for pH 5.5 (route “4”), 7.4 (route “5”) and 8.0 (route “6”) are shown in Figure 4a. In routes “4”, “5,” and “6”, the bands at ~30 kDa under reduced conditions are single, and no other bands were detected, indicating that their purities were high. The two bands at 26 kDa (faint/monomer) and 46 kDa (robust/dimer) under non-reduced conditions and the band at ~30 kDa (monomer) under reduced conditions for routes “4”, “5” and “6” are very similar with those observed in routes “1”, “2” and “3”.

The results of peptidase activity tests for cleavage of R-pNA by #7TR using size-exclusion chromatography are shown in Figure 4b. The catalytic activity of #7TR purified at pH 5.5 was low, while the activity purified at pH 7.4 and 8.0 was much higher than that of pH 5.5. This tendency was similar to that observed in previous experiments by cation exchange chromatography (Figure 3b). #7TR prepared at pH 8.0 exhibited the highest catalytic activity among all samples tested in this study.

#### 2.1.4. pH Dependency

The relation between pH and catalytic activity of #7TR (6.8 μM) was investigated, where #7TR was purified by size-exclusion chromatography (pH 7.4). In the tests, pH was varied by changing the reaction buffer as described in Materials and Methods. (Briefly, pH 5.5 was adjusted by sodium acetate buffer, pH 6.0 and 7.0 by phosphate buffer, and pH 8.0 and 9.0 by Tris-HCl buffer).

**Figure 5:**
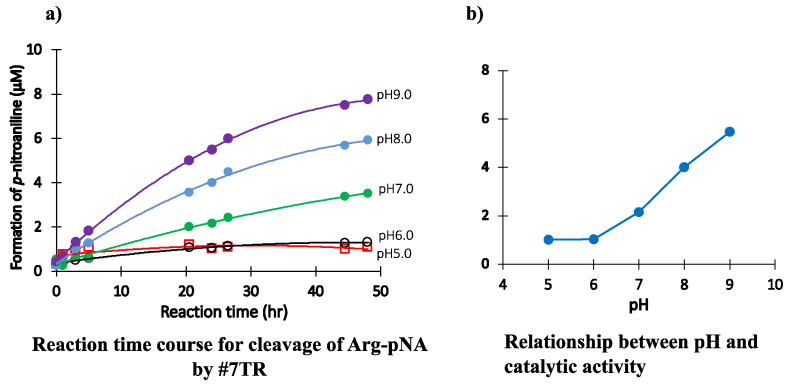
**pH dependency.** Investigation of pH dependency on the catalytic activity of #7TR in reaction buffers: (**a**) **Reaction profiles for each pH.** #7TR; 6.8 μM (purified by route”6” (pH 7.4)). Substrate; Arg-pNA of 200 μM, pH 5; acetate buffer, pH 6 and 7; phosphate buffer, pH 8 and 9; Tris-HCl buffer. (**b**) **Relationship between pH and catalytic activity.** For easy understanding, the relation between pH and the concentration of produced *p*-nitroaniline at 27 h of incubation was presented. \the isoelectric point of #7TR was 6.52.

The reaction time courses for each pH are shown in Figure 5a, where pH was varied from 5.0 to 9.0. The catalytic activity to cleave the R-pNA substrate was low at pH 5.0 and 6.0, whereas it was higher at pH 7.0, 8.0, and 9.0. In a basic condition, the catalytic activity increased in a pH-dependent manner. In contrast, under an acidic condition, the catalytic activity was hugely suppressed. In Figure 5b, the relation between pH and the concentration of produced *p*-nitroaniline at 27 h of incubation was presented for easy understanding. The substrate R-pNA was gradually cleaved over pH 7.0. As the isoelectric point of #7TR is 6.52, the purification pH, which is larger than the isoelectric point, might be important for revealing the intrinsic catalytic function.

#### 2.1.5. Effect of Other Components (Reagents)

##### NaCl

A salt, NaCl, is often used in the experiment of life sciences because of adjusting the osmotic pressure, stabilizing proteins etc. In all cases performed in the above purification experiments, NaCl was included (250 mM NaCl/Ni-NTA; 150 mM NaCl/ cation-exchange; 137 mM NaCl/size-exclusion). By using size-exclusion chromatography, we performed the #7TR purification without NaCl at pH 5.5 and 8.0.

**Figure 6:**
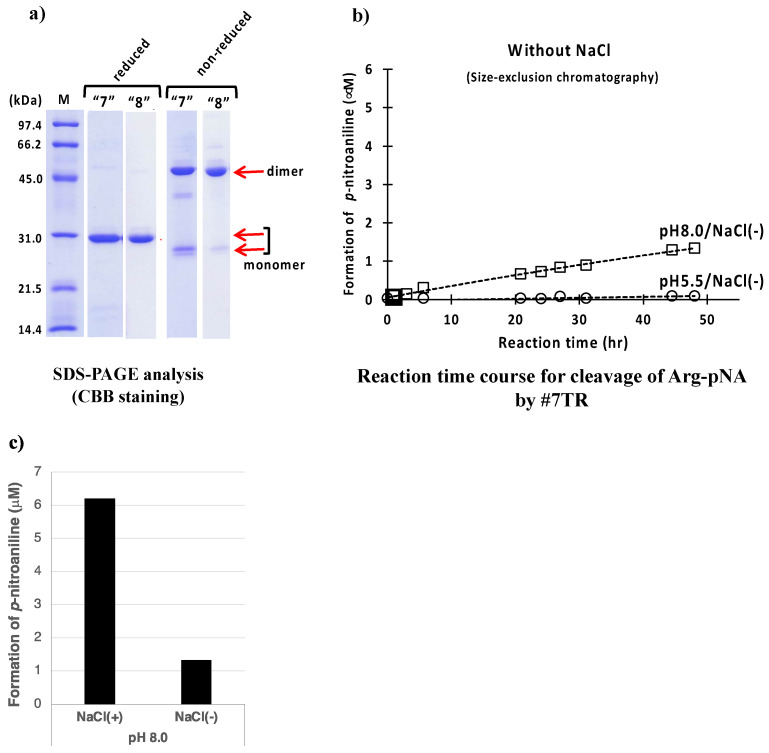
**Effect of NaCl.** (**a**) **SDS-PAGE analysis.** Bands were visualized by CBB staining. Under the reduced condition, only the ~30 kDa band corresponding to the monomer was detected as a single band. Bands other than the monomer band of the light chains were barely observed, suggesting that the #7TR light chains were highly purified. Under the non-reduced condition, bands at ~46 kDa corresponding to the dimer and at ~26 kDa corresponding to the monomer were detected. (**b**) **peptidase activity.** #7TR was purified without NaCl by route “7” at pH 5.5 and route “8” at pH 8.0. #7TR; 6.8 μM, Arg(R)-pNA; 200 μM. The catalytic activity of#7TR purified by route “8” at pH 8.0 without NaCl was low. The activity of #7TR purified by route “7” at pH 5.5 was extremely low. (**c**) **Comparison of the effect of NaCl.** The effect of NaCl is summarized to understand at a glance, where the catalytic activity measured at 48 h is compared.

The SDS-PAGEs at pH 5.5 are shown in routes “7” and “8” of Figure 6a. Under the reduced conditions, #7TR became a single band, indicating that it was highly purified. In the sample obtained from route “7” under non-reduced condition, the monomeric form was slightly richer than that of route “8”. Another faint band at ~40 kDa was detected between the dimer and monomer bands. 

The catalytic reactions for these samples are presented in Figure 6b. Both catalytic activities of #7TR purified at pH 5.5 and 8.0 without NaCl lowered by comparison with those with NaCl (see Figure 4b). In Figure 6c, the effect of NaCl is summarized to understand at a glance, suggesting that NaCl works to enhance the catalytic activity or keep the high activity.

##### Tween-20

**Figure 7:**
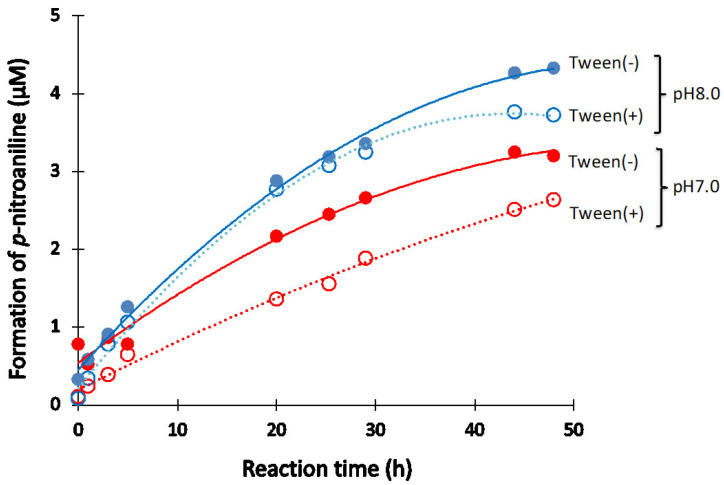
**Effect of Tween-20.** Effect of Tween-20 on the catalytic activity of #7TR purified by route “5” (pH 7.4) was investigated. #7TR; 6.8 μM, Arg-pNA; 200 μM, Tween-20; 2.5%.

Tween-20 is a surfactant is often used in immunoreaction experiments. Thus, the effect of Tween-20 on the catalytic activity of #7TR (6.8 μM) purified by route “5” was investigated under the reaction conditions at pH 7.0 and 8.0. Figure 7 presents the results. In the case of pH 7.0, it is clear that the Tween-20 (2.5%) lowered the catalytic activity. A similar effect was observed at pH 8.0 after more than 40 h of incubation. 

#### 2.1.6. Kinetics Using #7TR Purified by Route ”5” (pH 7.4) 

**Figure 8:**
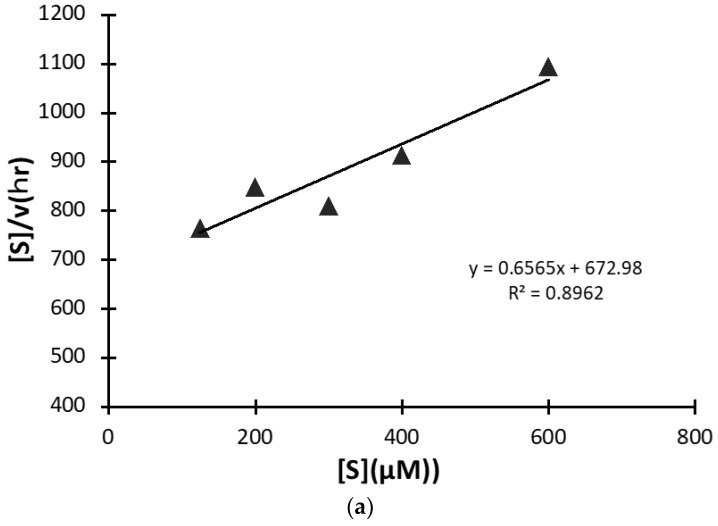

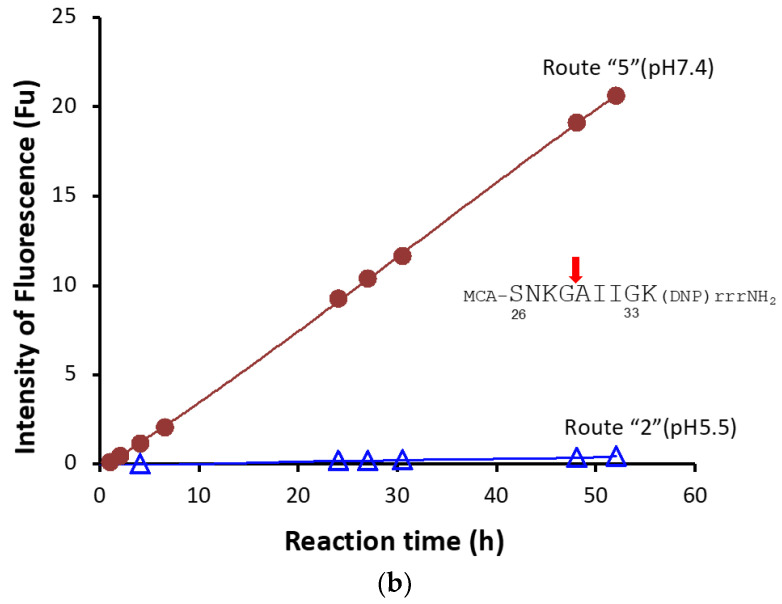
(**a**) **Kinetics by #7TR.** #7TR (purified by route”5” (pH 7.4)); 10 μM, Arg-pNA 125~600 μM. Reaction temperature: 37 °C. The values of kcat and Km were 2.5 × 10^−3^ min^−1^and 1.05 × 10^−4^M, respectively. (**b**) **Cleavage reaction for amyloid-beta (Aβ; 26–33) peptide.** #7TR (purified by route”5” (pH 7.4)); 10 μM. FRET-Aβ; 20 μM. Reaction temperature: 37 °C. #7TR has a characteristic feature to cleave the peptide bond between Gly29 and Ala30 of Aβ peptide.

A kinetic study was carried out using #7TR purified by route”5” (pH 7.4), where #7TR was kept at 10 μM and concentrations of the substrate R-pNA were varied from 125 to 600 μM. The obtained result of the Hanes-Woolf plot is presented in Figure 8a. It shows a linear relationship between [S] vs [S]/V. Thus, #7TR obeys the Michaelis-Menten equation as an enzymatic reaction. The values of kcat, Km, and kcat/Km were 2.5 × 10^−3^ min^−1^, 1.05 × 10^−4^ M, and 2.38 × 10 min^−1^M^−1^, respectively. These values are comparable with those of Durova et al. [16].

#7TR light chain has a characteristic feature to cleave the substrate of R-pNA as well as FRET-Amyloid-beta (Aβ) peptide (7-MCA-SNKGAIIG-K(DNP)rrrNH_2_). Thus, the cleavage reaction for FRET-Aβ was carried out using the #7TR purified with route “5”, where 20 μM of FRET-Aβ substrate and 10μM of #7TR were used. The reaction time course is shown in Figure 8b. The cleavage of FRET-Aβ gradually proceeded up to 55 h of incubation in a time-dependent manner, where the peptide bond between G29 and A30 was digested [36].

### 2.2. H34 Light Chain

To ascertain whether or not the similar phenomenon described in the previous section is observed in general, another catalytic antibody light chain H34 (aa sequence: see [37]) belonging to Subgroup I was used, as shown in the following experiments.

H34 catalytic antibody light chain was prepared using three different purifications (Purification (I); Ni-NTA at pH 8.0, Purification (II); cation-exchange at pH 5.5, and Purification (III); size-exclusion at pH 7.4), based on the previous results.

The catalytic activity of H34 was estimated using the FRET-PD1 peptide (aa 123rd–140th ((7-methoxycoumarin-4-yl)acetyl [7-MCA]-^123^GAISLAPKAQIKESLRAE^140^-K(2,4-dinitrophenyl [DNP])-NH_2_; this sequence is the epitope of Nivolumab (anti-PD-1 mAb); FRET is formed by adducting MCA at the N-terminal and DNP at the C-terminal. Using the FRET-PD1 substrate (25 μM), the cleavage reactions of H34 (0.25~5 μM) prepared by the three different purifications were examined.

#### 2.2.1. Purification (I) at pH 8.0 (Ni-NTA Chromatography)

**Figure 9:**
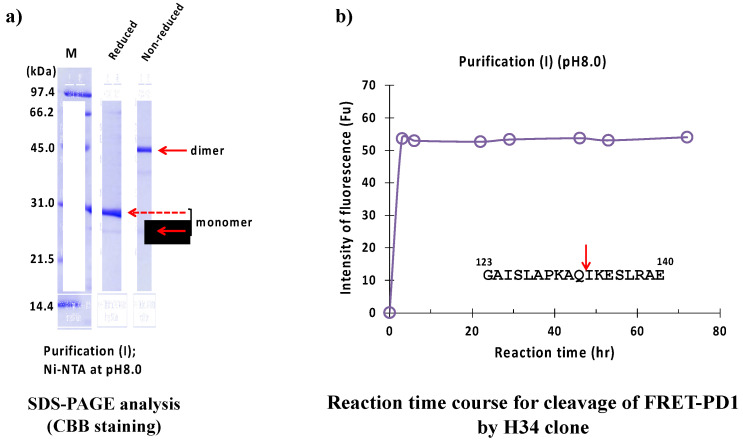
SDS-PAGE and peptidase activity of H34 prepared by purification (I) at pH 8.0. (**a**) SDS-PAGE analysis. (**b**) PD-1 cleavage reaction. H34; 5 μM, FRET-PD1; 20 μM.

The result of the SDS-PAGE analysis is shown in Figure 9a, where a monomer band under reduced conditions appeared at approximately 29 kDa. The band at around 45 kDa and a faint band at around 25 kDa under non-reduced conditions corresponded to the dimer and monomer of the H34 light chain, respectively. The monomer band was very faint, while the dimer was visible, which suggests that the dimer is dominant in the solution. Figure 9b shows the reaction time course of H34 purified with purification (I) (pH 8.0). The FRET-PD1 substrate was rapidly hydrolyzed within 2 h of incubation, indicating that #7TR showed high catalytic activity. The cleavage peptide bond was between Gln132 and Ile133 of the PD-1 peptide [33].

#### 2.2.2. Purification (II) at pH 5.5 (Cation-Exchange Chromatography)

**Figure 10:**
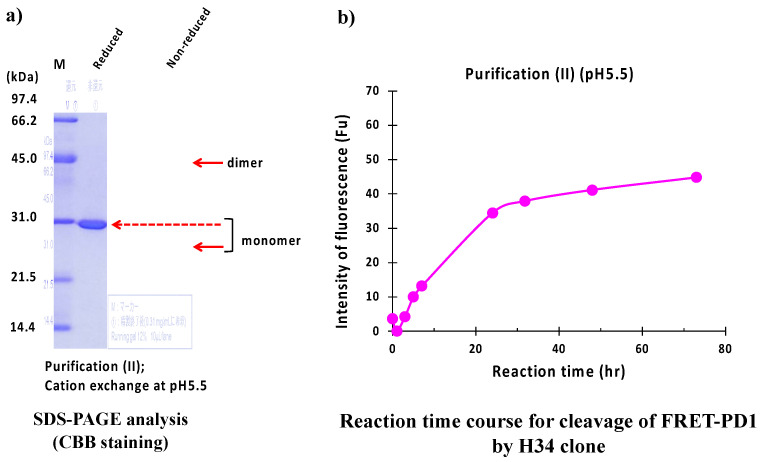
SDS-PAGE and peptidase activity of H34 prepared by purification (II) at pH 5.5. (**a**) **SDS-PAGE analysis.** (**b**) **PD-1 cleavage reaction.** H34; 5 μM, FRET-PD1; 20 μM.

The result of the SDS-PAGE analysis is shown in Figure 10a. where the identical monomer was observed at 29 kDa under reduced conditions. The visible band at 45 kDa and a faint band at 25 kDa under non-reduced conditions were dimer and monomer, respectively. 

Figure 10b displays the reaction time course of H34 purified with purification (II) (pH 5.5). In this case, the concentration of product pNA was gradually increased up to 20 h of incubation in a time-dependent manner, and then it reached the plateau. Note that the cleavage of the FRET-PD1 substrate was considerably suppressed using #7TR prepared with pH 5.5.

#### 2.2.3. Purification (III) at pH 7.4 (Size-Exclusion Chromatography)

**Figure 11:**
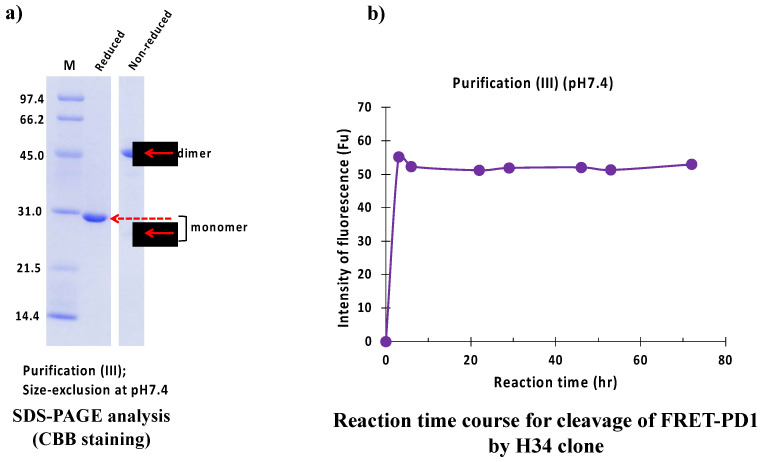
SDS-PAGE and peptidase activity of H34 prepared by purification (III) at pH 7.4 (**a**) SDS-PAGE analysis. (**b**) PD-1 cleavage reaction. H34; 5 μM, FRET-PD1; 20 μM.

The result of the SDS-PAGE analysis is shown in Figure 11a. Under reduced conditions, the monomer band was detected at 29 kDa. Under non-reduced conditions, a faint band at 25 kDa and 45 kDa represented monomer and dimer, respectively. 

Figure 11b represents the reaction time course of H34 purified with purification (III) (pH 7.4). H34 cleaved FRET-PD1 in a short time (within 2 h). 

#### 2.2.4. Comparison of Ni-NTA (I) and Size-Exclusion (III) Chromatography

**Figure 12:**
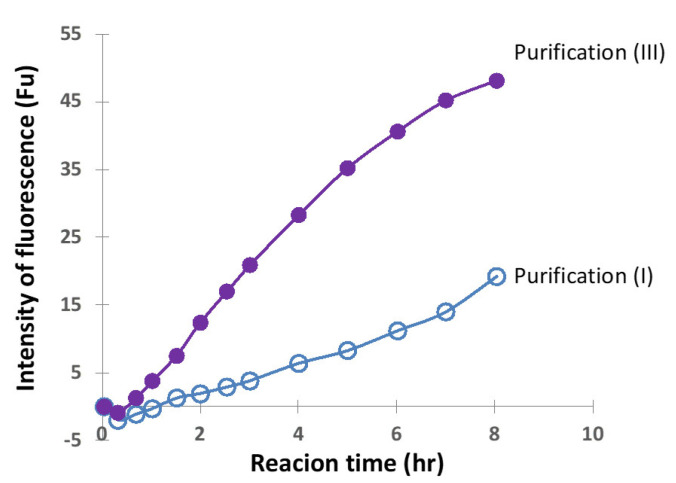
**Comparison of purification (I) and (III).** FRET-PD1; 25 µM, H34; 0.5 µM. The H34 (0.5 µM) prepared by Purification (III) (pH 7.4) almost hydrolyzed the FRET-PD1substrate within 8 h. The H34 prepared by purification (I) (pH 8.0) did not hydrolyze the substrate by half.

In order to precisely compare the above two cases, the concentration of H34 was decreased from 5 µM to 0.5 µM while keeping the FRET-PD1 substrate concentration at 25 µM. The reaction profiles for purification (I) and (III) are presented in Figure 12. The FRET-PD1 substrate was almost hydrolyzed within 8 h by H34 purified by (III). At the same time, H34 by purification (I) did not hydrolyze the substrate by half, indicating that purification (III) is better than (I), suggesting that size-exclusion chromatography was the best in the experiments used in this study. 

#### 2.2.5. Kinetics

**Figure 13:**
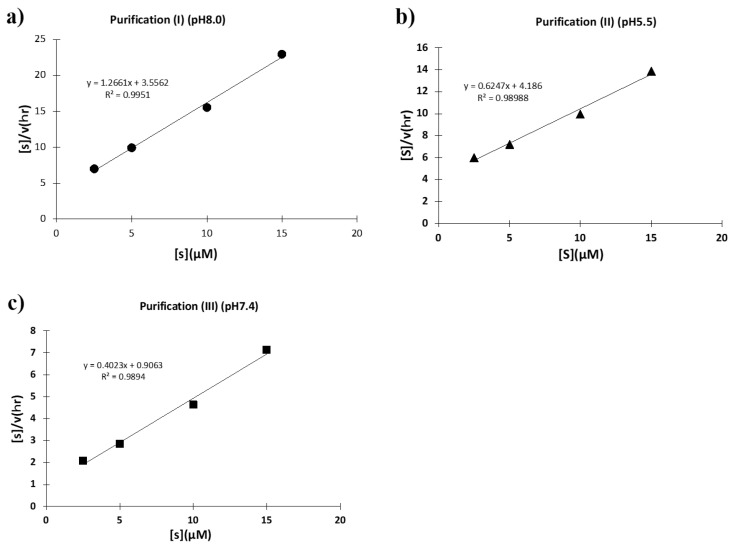
**Kinetics by H34.** The concentration of FRET-PD1 substrate was varied from 2.5 to 15 µM at 37 °C keeping the concentration of H34 at 5 μM or 0.25 μM in TGT buffer (pH 7.7). [S]: concentration of FRET-PD1 substrate. [V]: initial rate of the cleavage reaction. (**a**) **H34 (0.25 μM) from Purification (I) (pH 8.0)**: The Hanes–Woolf plot demonstrates that the cleavage reaction by the purification (I) H34 obtained by purification (II) fits the Michaelis–Menten kinetics equation. From the plot, kcat and Km were 5.5 × 10^−2^ min^−1^ and 3.24 × 10^−6^ M, respectively. (**b**) **H34 (5.0 µM) from Purification (II) (pH 5.5)**: From the Hanes–Woolf plot, kcat, and Km were 5.8 × 10^−3^ min^−1^ and 7.87 × 10^−6^ M, respectively. (**c**) **H34 (0.25 μM) from Purification (III) (pH 7.4).** From the Hanes–Woolf plot, kcat, and Km were 1.8 × 10^−1^ min^−1^ and 3.07 × 10^−6^ M, respectively.

To investigate what caused these different catalytic activities, kinetic studies were performed using the synthetic substrate FRET-PD1. For each case, Hanes–Woolf plots ([S]/v vs. [S]) were taken. First, the plots for the H34 sample purified at a pH of 8.0 (purification (I)) is shown in Figure 13a, where a good linear relationship was observed, indicating that the reaction obeyed the Michaelis-Menten equation and the reaction was enzymatic. The kcat and Km values were 5.5 × 10^−2^ min^−1^ and 3.24 × 10^−6^ M, respectively. The kcat/Km value (catalytic efficiency) was 1.7 × 10^4^ min^−1^ M^−1^. The kcat was higher than that of the sample purified at a pH of 5.5 (purification (II)) by a factor of 22.9-fold, which is described in the following. Second, the H34 sample purified at a pH of 5.5 (purification (II)) was examined. As shown in Figure 13b, a good linear relationship was observed in this case, too. The kcat, Km, and kcat/Km values were 5.8 × 10^−3^ min^−1^, 7.87 × 10^−6^ M, and 7.4 × 10^2^ min^−1^M^−1^, respectively. Third, Figure 13c shows the H34 sample purified at a pH of 7.4 (purification (III)). The kcat and Km values were 1.8 × 10^−1^ min^−1^ and 3.07 × 10^−6^ M, respectively. The kcat/Km value was 1.7 × 10^4^ min^−1^M^−1^, which was higher than that of the sample of purification (II) by a factor of 79.7-fold. Thus, the sample purified in a basic condition exhibited an intrinsic catalytic ability, whereas the catalytic function was suppressed when purified in an acidic condition.

## 3. Discussion

The activity of enzymes and catalytic antibodies depends on many factors of preparation conditions, including the purification method, pH, salt, and metal ions used [32,34,36,38,39,40,41,42]. The most preferable preparation method and condition to exhibit the high and/or intrinsic catalytic function must be explored and determined for future therapeutic applications. Based on this strategy, we undertook systematic experiments to investigate different purification methods and conditions, as stated above. 

Table S1 represents the summary of the data obtained for #7TR purification in this study. First, route “1” was performed to obtain the sample of #7TR purified by Ni-NTA affinity chromatography under a basic condition (pH 8.0). Though the sample was not subjected to the second purification, the purity was high. The #7TR showed moderate catalytic activity to cleave the R-pNA substrate (Figure 2b). 

The samples of routes “3”, “5” and “6” were purified under the basic conditions at pH 8.0, 7.4, and 8.0, respectively, in the second purification step (“3”; cation exchange, “5” and ”6”: size exclusion). These three samples exhibited high or moderate peptidase activity. The sample of route “6” showed the highest activity out of all tested in this study. Conversely, the samples purified by routes “2” (cation exchange) and “4” (size-exclusion) under an acidic condition (pH 5.5) in the second purification step showed low catalytic activity. Although all samples were dialyzed against PBS (pH 7.4) at the final stage, huge differences were observed depending upon the preparation conditions. The optimum condition should be important to present the intrinsic catalytic activity. CD spectra between route”2” (pH 5.5) and “3” (pH 8.0) and between ”2” (pH 5.5) and “6” (pH 8.0) did not show a clear difference between them. It is considered that the reduction of catalytic activity at low pH was caused by the protonation of the aspartate participating in the catalytic site. 

The isoelectric point of #7TR is 6.52. Interestingly, the high catalytic activity of #7TR was exhibited at higher pH than the isoelectric point. In contrast, the catalytic activity decreased at lower pH than the isoelectric point (Figure 5b). Planque et al. [34] reported a similar phenomenon for the catalytic antibody in their study, where both acid-purified aIgV and neutral-purified nIgV were used. Sharma et al. [36] described the optimum pH as 8.0 for the catalytic activity of the human antibody light chain. In addition, the pH dependency observed in this study resembles the results reported by Matsuura et al. [38]. Note that the purification conditions had a definitive influence on the catalytic activity. The effect of pH was substantially important. The catalytic antibody should be prepared in a basic condition with pH > 7.0 to display the high and/or intrinsic catalytic activity. In the case that the triad (Asp-Ser-His) is concerned with the catalytic site, the above idea is also supported. The aspartate would be protonated in acidic pH; hence, it cannot draw the proton from the histidine and therefore, serine will not be able to make the nucleophilic attack on the scissile bond. This will cause the reduction of the catalytic activity.

Regarding the effect of NaCl, the experiments of “4” (NaCl(+)) and “7” (NaCl(-)) at pH 5.5 and routes “6” (NaCl(+)) and “8” (NaCl(-)) at pH 8.0 were performed in size-exclusion chromatography. By comparing Figure 4a with Figure 6a, route “6” showed a six-fold higher catalytic activity than route “8.” The same tendency was also observed in the case of routes ”4” and “7”. The presence of NaCl is advantageous for catalytic activity. NaCl would be necessary to maintain preferable conformation to display the catalytic activity.

To ascertain whether or not the above results are common, we experimented using another catalytic light chain, H34 (belonging to Subgroup I), which can enzymatically cleave the FRET-PD1 substrate [37] and was prepared by Purification (I) (pH 8.0; Ni-NTA), Purification (II) (pH 5.5: cation-exchange), and Purification (III) (pH 7.4; size-exclusion). The samples by Purification (I) and (III) showed high catalytic activity to cleave the FRET-PD1 substrate (Figure 9b and Figure 11b), whereas the sample purified by Purification (II) showed low activity (Figure 10b). The results for the H34 catalytic antibody were similar to those observed for #7TR. The order of catalytic activity of H34 is Purification (III) > Purification (I) >> Purification (II). It is concluded that the catalytic antibody light chain should be purified under a high pH (over 7.0) but not a low pH (below 6.0).

Regarding the kinetic studies using the H34 catalytic light chain, the kcat, Km, and kcat/Km values for the three samples are listed in Table S2 and the data from the references. For Purification (II) (acidic condition: pH 5.5), the kcat was 5.8 × 10^−3^ min^−1^, whereas those for Purification (I) (pH 8.0) and (III) (pH 7.4) were 5.5 × 10^−2^ min^−1^ and 1.8 × 10^−1^ min^−1^, respectively. The kcat of Purification (I) was higher than that of Purification (II) by a factor of 10, and the kcat of Purification (III) was higher than that of Purification (II) by a factor of 30. Interestingly, the kcat value was drastically changed depending on the preparation pH. On the other hand, the Km values were almost consistent (from 3.1 to 7.9 × 10^−6^ M), suggesting that the ability recognize the antigen is kept constant. Anyhow, the structure of the catalytic site to cleave PD-1 might be modulated during the progress of the chromatography.

With respect to the kinetic study relating to pH, Durova et al. [16] reported a study where kcat was found to be 1.55 × 10^−3^ min^−1^ and Km = 5.3 × 10^−5^ M using an L12 light chain for the cleavage of the Pro-Phe-Arg-MCA substrate, where the sample was eluted at a pH of 2.6 and immediately neutralized with 1.0 M Tris-HCl (pH 9.0), followed by dialysis against the phosphate buffer (pH 8.0). In this case, the catalytic activity (kcat) to cleave the substrate is comparable to that observed in Purification (II) at pH 5.5 in this study. Planque et al. [34] also reported on the kinetic values using a recombinant antibody light chain (aIgV) eluted at a pH of 5.0. The kat and Km were 3.0 × 10^−1^ min^−1^ and 8.0 × 10^−5^ M, respectively. The kcat/Km was 3.8 × 10^3^ min^−1^ M^−1^, which was higher than that of Purification (II) but lower than that of Purification (I). The kcat/Km value of Purification (III) was higher than that of aIgV by a factor of 15, implying that the catalytic activity of aIgV will be improved if it is prepared under a basic condition.

Since 1989 by Paul et al., many catalytic antibodies have been produced, where various preparation methods and conditions were employed. As the catalytic activity is changeable depending on the preparation methods and conditions, analysis obtaining the highly active and reproducible methods and conditions has been required so far. The knowledge obtained in this study contributes to the development of catalytic antibodies for numerous applications.

## 4. Materials and Methods

### 4.1. Reagents

Chemical reagents of Tris, glycine, CuCl_2_·2H_2_O, KCl, Na_2_HPO_4_·12H_2_O, NaCl, KH_2_PO_4_, EDTA·2Na, and IPTG were purchased from Wako Pure Chemical Industries Ltd., Osaka, Japan (Guaranteed Reagent). The synthetic substrate peptidyl-pNA, Arg-pNA, was purchased from Peptides Institute Inc., Osaka, Japan. Tryptone and yeast extract were purchased from Becton-Dickinson and Company, Sparks, MD, USA.

### 4.2. Synthesis of FRET Substrates

The FRET-Aβ substrate (7-MCA-SNKGAIIGK(DNP)rrr-NH_2_; Aβ aa26–33) and FRET-PD1 peptide (7-MCA-GAISLAPKAQIKESLRAE-K(DNP)-NH_2_; PD1 aa123–140) were synthesized on a solid support using Fmoc/tBu strategy on the Rink amide resin as previously reported [37]. Briefly, the removal of the Fmoc group was carried out with 20% piperidine in dimethylformamide, whereas the chain elongation was achieved with standard HBTU/HOBt chemistry using three equivalents of protected amino acids or 7-MCA. After completing the synthesis, the protected peptide resin was treated with TFA/phenol/H_2_O/thioanisole/1,2-ethanedithiol (82.5:5:5:2.5, *v*/*v*/*v*/*v*) mixture. The crude material obtained was purified by HPLC. The molecular weight and chemical structures of FRET peptides were confirmed by MS.

### 4.3. Amplification of DNA Fragments Encoding Light Chains

Preparations of the #7TR and H34 genes were obtained in accordance with that described in the references [35,43].

### 4.4. Sequencing 

The #7TR and H34 clones were sequenced with an ABI 3730xl Analyzer (Applied Biosystems, Waltham, MA, USA) using ABI BigDye™ Terminator v3.1 Cycle Sequencing Kits. GENETIX Ver. 8 (GENETIX, Tokyo, Japan) software was used for sequence analysis and deduction of amino acid sequences.

### 4.5. Culture, Recovery, and Purification

The transformant was grown at 37 °C in 1 L of Luria-Bertani medium containing 100 μg/mL ampicillin to an A600 nm of 0.6 and then incubated with 0.01 mM IPTG for 20 h at 18 °C. Cells were harvested by centrifugation (3500× *g*, 4 °C, 10 min) and then resuspended in a 100 mL solution of 250 mM NaCl, 25 mM Tris-HCl, pH 8.0). The cells were lysed by ultra-sonication three times for 2 min each in an ice bath, followed by centrifugation (21,475× *g*, 4 °C, 20 min). The expressed human light chain was recovered as the supernatant.

### 4.6. Ni-NTA Column Chromatography (Route “1”)

The supernatant was first subjected to Ni-NTA column chromatography (Takara, Otsu, Japan) equilibrated with 25 mM Tris-HCl, pH 8.0, containing 250 mM NaCl. Elution was performed by increasing the concentration of imidazole from 0 and/or 30 to 300 mM. The detailed procedure, chromatography, and SDS-PAGE analysis are shown in Figure S1. After the Ni-NTA column chromatography was completed, an aliquot of a solution of 50 mM CuCl_2_ (1.25 eq for the light chain) was added into the eluent (this is important to make a uniform (dimer) structure), based on the calculation that the absorbance of A600 nm of 1.0 in UV/VIS was regarded as ~1 mg/mL (40 μM light chain), and allowed to react for 16 h. In all purification steps mentioned below, the mixture of the eluted solution and copper ion was made and applied to each purification. 

The solution was concentrated to 3–6 mg/mL by the filter (Amicon ultra-10000; Millipore, Burlington, MA, USA, PEF-UFC80196, Lot. R5AA49895) and the final concentration of 50mM of EDTA was added and allowed for 1 h at 4 °C, followed by the dialysis against PBS (2 L) twice. If aggregates were found in the solution, they were removed by centrifugation (21,475× *g*, 20 min at 4 °C), and the supernatant was sterilized by a filter (0.22 µm; Millex-GV, 13 mm, REF-SLGVJ13SL, Lot. RAA48083, PVDF) and it was stored at 4 °C or frozen.

### 4.7. Cation Exchange Chromatography (Route “2 & 3”) 

#### 4.7.1. Purification at pH 5.5 (Route “2”) 

The solution, including the light chain and copper ion after Ni-NTA column chromatography, was dialyzed against a 50 mM Na acetate buffer, pH 5.5, overnight and subjected to cation exchange chromatography on an SP-5PW column (Tosoh, Tokyo, Japan) with a gradient of NaCl solution (from 0.1 to 0.5 M) on the purification apparatus (AKTA System; GE Healthcare, Tokyo, Japan). The eluent was dialyzed against a buffer of 20mM Tris-HCl/150mM NaCl (pH 8.5) for 15–20 h, followed by solution concentration with the filter (Amicon ultra-10000). The final concentration of 50mM of EDTA was added and allowed for 1 h at 4 °C, followed by the dialysis against PBS (2 L; pH 7.4) twice.

#### 4.7.2. Purification at pH 8.0 (Route “3”)

The dialysis of the solution, including the light chain and copper ion, was performed against 50 mM Tris-HCl (pH 8.0). It was subjected to cation-exchange chromatography using a column of SP-5PW (TOSOH, Japan) eluted with a Tris-HCl buffer (pH 8.0) on the purification apparatus (AKTA system, GE Healthcare, Japan, Tokyo). The eluent (as a flow-through) was recovered and submitted to dialysis against 20 mM Tris-HCl/150 mM NaCl buffer (pH 8.5) for about 17 h, followed by concentrating the solution using the filter (Amicon ultra10000). Other procedures were the same as stated in the above pH 5.5.

### 4.8. Size-Exclusion Chromatography (Route “4–8”) 

#### 4.8.1. Purification at pH 5.5 (Route “4”)

The solution, including the light chain and copper ion after Ni-NTA column chromatography, was dialyzed against a 50 mM Na acetate buffer with 137 mM NaCl (pH 5.5) overnight and subjected to size-exclusion chromatography (column; HiLoad^TM^16/60 Superdex^TM^ 200 pg (GE healthcare)) on the AKTA system, with a 50 mM Na acetate buffer including 137 mM NaCl (pH 5.5) used as the eluent solvent.

#### 4.8.2. Purification at pH 7.4 (Route “5”) 

PBS (pH 7.4) was used instead of 50 mM Na acetate buffer in the dialysis buffer as well as the eluent solvent.

#### 4.8.3. Purification at pH 8.0 (Route “6”) 

All procedures were the same as performed in Route “4” except for the dialysis buffer and the eluent solvent. In this route, a 50 mM Tris-HCl, including 137 mM NaCl (pH 8.0), was used for the dialysis of the solution of the light chain and copper ion

#### 4.8.4. Purification at pH 5.5 (Route “7”) 

All procedures were the same as performed in Route “4” except for the dialysis buffer and the eluent solvent. In route “7”, NaCl was not contained in the 50 mM Na acetate buffer for the dialysis as well as in the eluent solvent.

#### 4.8.5. Purification at pH 8.0 (Route “8”) 

A 50 mM Tris-HCl without NaCl (pH 8.0) was used instead of a 50 mM Tris-HCl with 137 mM NaCl (pH 8.0) in route “8”.

All solutions stated above were concentrated to 2–6 mg/mL and dialyzed against PBS (pH 7.4) twice, first for 6 h and then for 16 h. After filtration with the 0.2 µm filter, the solution was stored at 4 °C or frozen. Protein concentrations were determined using the DC protein assay kit (Bio-Rad, Hercules, CA, USA) by a Lowry method.

### 4.9. Cleavage Assays

To avoid contamination in cleavage assays, most glassware, plastic-ware, and buffer solutions used in this experiment were sterilized by heating (180 °C, 2 h), autoclaving (121 °C, 20 min), or filtration through a 0.20-μm sterilized filter, as much as possible. Most of the experiments were performed in a biological safety cabinet to avoid airborne contamination. 

#### 4.9.1. Arg(R)-pNA Substrate

Cleavage of the amide bond linking *p*-nitroanilide to the C-terminal aa in R-pNA substrates (Peptides Institute Inc., Osaka, Japan) was measured at 37 °C in a glycine/ Tris buffer containing 0.025% Tween 20 (TGT buffer; pH 7.7) in 96-well plates (96-well plate/353075, Becton-Dickinson, Sparks, MD, USA). The purified light chain (20 μL) was mixed with 180 μL of a synthetic substrate, R-pNA. The final concentrations of the light chain and the substrate were 6~10 μM and 200 μM, respectively. Para-nitroaniline formed from the substrate catalyzed by the light chains was detected by the measurement of absorbance at 405 nm, while 620 nm was employed as the reference using a microplate reader (Scanlt 3.1 for Multiskan FC, ThermoFisher Scientific, Waltham, MA, USA). The peptidase activity of catalytic antibodies was estimated from the concentration of formation of *p*-nitroaniline.

#### 4.9.2. FRET-Aβ and FRET-PD1 Substrates

FRET-Aβ or FRET-PD1 substrates (25 µM) were incubated with #7TR or H34 light chain (5 µM) in TGT buffer containing 0.02% NaN_3_ at 37 °C. Fluorescence was measured periodically on the Fluoroskan Ascent (λ_ex_ = 320 nm and λ_em_ = 405 nm: Thermo Fisher Scientific Oy, Vantaa, Finland). All measurements were done in duplicate. 

### 4.10. Kinetics

#### 4.10.1. Arg(R)-pNA

The concentration of the #7TR light chain was fixed at 10 µM, and that of the R-pNA substrate was varied from 125 to 600 µM at 37 °C in the TGT buffer (pH 7.7). The concentration changes of R-pNA substrate within 10% conversion after mixing the #7TR light chain and the substrate was regarded as the initial rate of the reaction.

#### 4.10.2. FRET-PD-1

The concentration of the H34 light chain was fixed at 5 µM (for purification (II)) or 0.25 µM (for purification (I) and (III)), and that of the FRET-PD1 substrate was varied from 2.5 to 60 µM at 37 °C in the TGT buffer (pH 7.7). The concentration changes of the FRET-PD1 substrate within 10% conversion after mixing the H34 light chain and the substrate was regarded as the initial rate of the reaction.

## 5. Conclusions

We can obtain the highly active catalytic antibody with high reproducibility by considering the following points:

(1) The catalytic antibody should be prepared under a basic condition. In contrast, the catalytic activity is hindered or lost when prepared under an acidic condition. The appropriate pH range for the preparation was from 7.0 to 9.0. The low catalytic activity under the acidic pH is caused by the protonation of the aspartate participating in the catalytic site. Another possibility is not excluded, where the conformational structure of the catalytic site may be modulated during the progress of chromatography.
(2) The presence of NaCl works to enhance or keep the high catalytic activity.
(3) Surfactant of Tween-20 works to decrease the catalytic activity a little.
(4) Size-exclusion chromatography is better than cation exchange chromatography.

With the utilization of these points, the development of catalytic antibodies can be accelerated for application to therapy etc.

## Data Availability

The data that supports the results of this study are included in the article and its Supplementary Materials.

## Supplementary Materials

The supporting information can be downloaded at: https://www.mdpi.com/article/10.3390/ijms232214351/s1.

## Abbreviations

aa, amino acid; AMC, 7-amino-4-methylcoumarin; au, arbitary unit; CBB, Coomassie brilliant blue; DNP: 2,4-dinitrophenyl; eq, equivalent; FRET, Förster resonance energy transfer; IPTG, isopropyl-β-D-thiogalactopyranoside; mAb, monoclonal antibody; 7-MCA, (7-methoxycoumarin-4-yl)acetyl; MS, mass spectroscopy; PBS, phosphate buffered saline; PD1, Programmed cell death-1; TGT, 50mM/Tris-100mM/Glycine-Tween-20 buffer; TFA, trifluoroacetic acid.
