# Supplementary material for: Obtaining Highly Active Catalytic Antibodies Capable of Enzymatically Cleaving Antigens
Source: Int J Mol Sci. 2022 Nov 18;23(22):14351. doi: 10.3390/ijms232214351 (PMC9697424; doi:10.3390/ijms232214351)
Supplement: Supplementary file 1 [file ijms-23-14351-s001.zip › ijms-1999035-supplementary.pdf]

Supplemental Figures and Tables

Figure S1 AA sequences for #7TR and H34 light chains

a) #7TR human catalytic antibody light chain

b) H34 human catalytic antibody light chain

Figure S2 Chemical structure of FRET adducted peptide substrates

For each peptide, MCA (4-methyl-coumaryl-7-amide; Fluorescent reagent) was adducted at N-terminal and DNP (2,4-dinitrophenyl; Quenching reagent). Lysine was inserted in order to bind with DNP.

a)FRET-Aβ substrate; FRET was adducted for the the peptide from 26 to 33 of Aβ.

b)FRET-PD1 substrate; FRET was adducted for the peptide from 123 to 140 of PD-1 (this region is the epitope of anti PD-1 mAb, Nivolumab).

Figure S3 Procedure, chromatography and SDS-PAGE analysis in 1st purification step (Ni-NTA column chromatography)

Amino acid sequences of #7TR and H34 human light chains

a) **#7TR**:Subgroup II of human kappa light chain (Kabat's classification)
MDVVMTQSPLSLPVTPGEPASISCRSSQSLLHSNTRNYLDWYLQKPGQSPQLLIYLGSNRAS
GVPDRFSGSGSGTDFTLKISRVEAEDVGVYYCMQALQTPRTFGQGTKVEIKRTVAAPSVFIFP
PSDEQLKSGTASVVCLLNNFYPREAKVQWKVDNALQSGNSQESVTEQDSKDSTYSLSSTLTL
SKADYEKHKLYACEVTHQGLSSPVTKSFNRGEC(LEHHHHHH)-----MW=25170.8

b) **H34**:Subgroup I of human kappa light chain (Kabat's classification)
MDIQMTQSPSTLSASVGDRVTITCRASQSISSWLAWYQQKPGKAPKVLIYKASTLESGVPLR
FSGSGSGTEFTLTISSLQPDDFATYYCQQYSTYRTFGQGTKVEIKRTVAAPSVFIFPPSDEQLKS
GTASVVCLLNNFYPREAKVQWKVDNALQSGNSQESVTEQDSKDSTYSLSSTLTLSKADYEKH
KVYACEVTHQGLSSPVTKSFNRGEC(LEHHHHHH)-------MW=24575.1

Red: CDR-1

Blue: CDR-2

Green: CDR-3

Methionine at 0th position is adducted because of insertion of a restriction enzyme site (*NcoI*).

(LEHHHHHH) is adducted because of insertion of His-tag.

Figure S1a and S1b

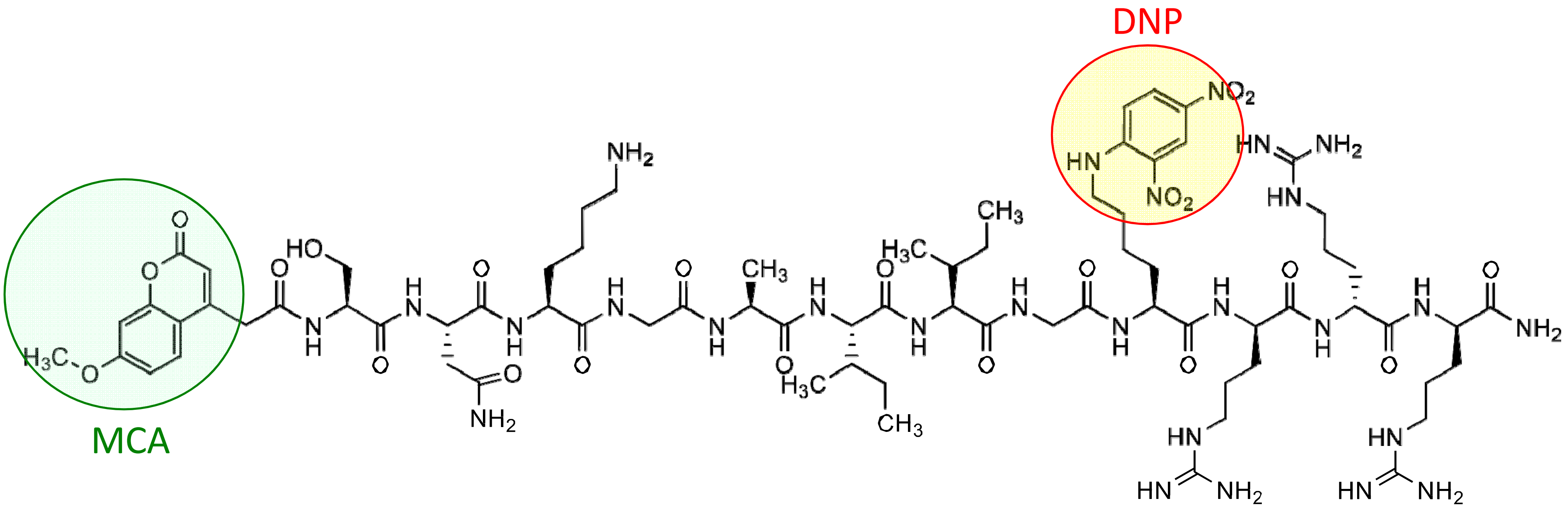

7-MCA-SNKGAIIG-K(DNP)rrrNH_2

26 33

Figure S2a

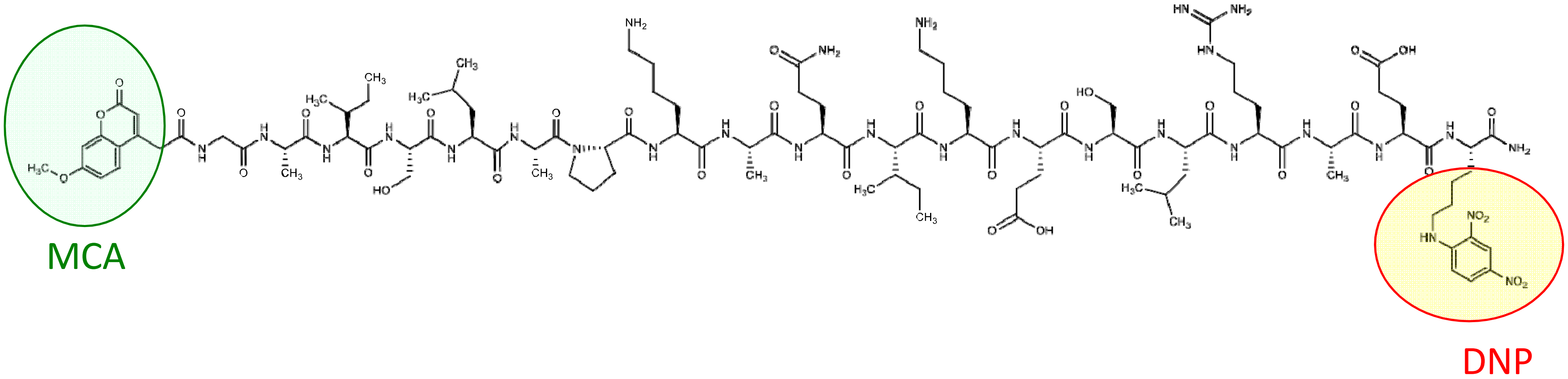

7-MCA-GAISLAPKAQIKESLRAE-K(DNP)-NH_2

Figure S2b

First purification step (Ni-NTA Column chromatography)

【Procedure】

Ni-NTA Column chromatography
Column; Ni-NTA agarose gel(QIAGEN)
Buffer; 250 mM NaCl, 25 mM Tris-HCl,pH8.0
Imidazole conc.;30-300 mM

← 1.25 eq $CuCl_2$ addition. Incubation overnight

↓

Concentration

← 50 mM EDTA addition. Incubation 1 h

↓

Dialysis for PBS

↓

Filtration (0.2µM). Stored at 4℃

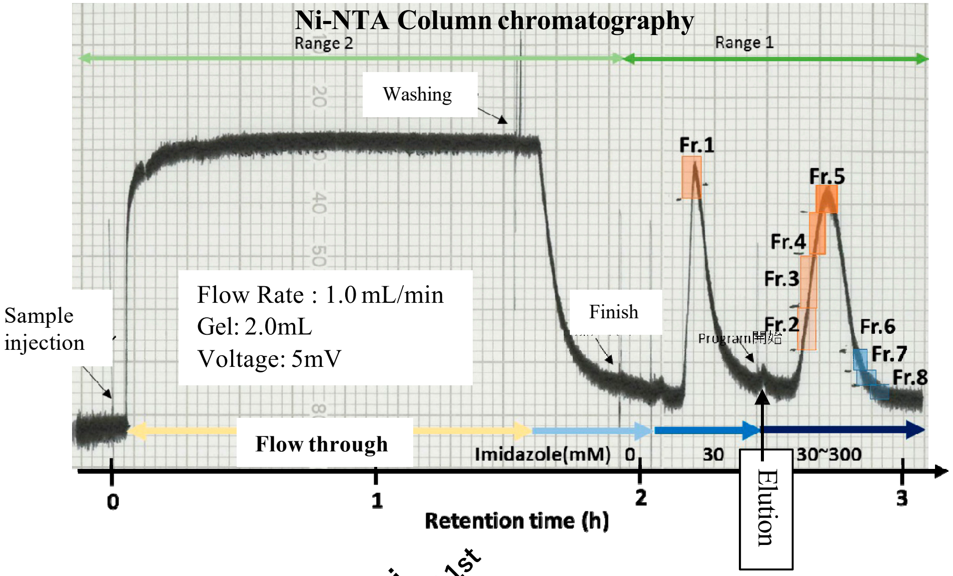

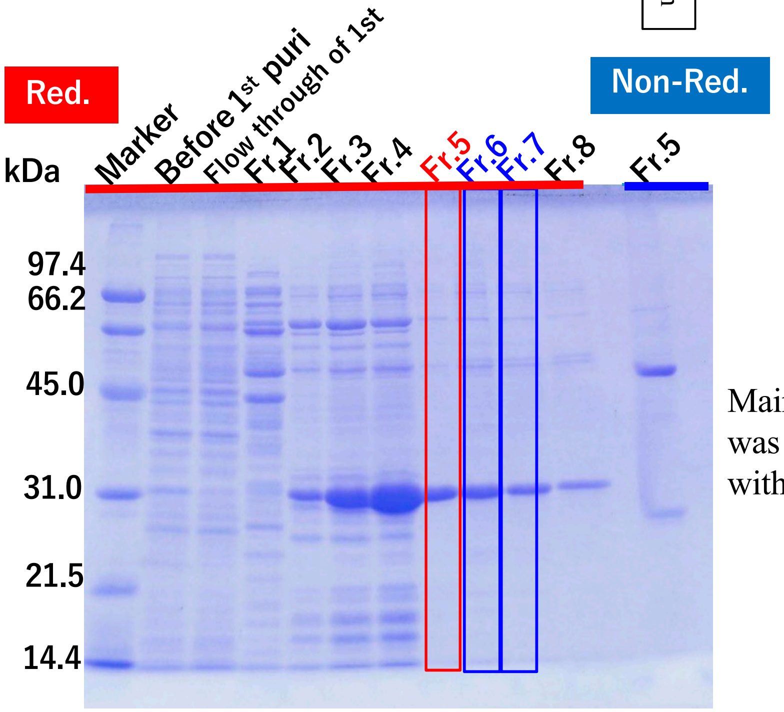

SDS-PAGE

Main fraction (Fr. 5) was recovered along with Fr. 6 and 7

Fr.5: Main fraction (applied with 1/10 dilution)

Figure S3

Table S1. Summary for preparations for #7TR light chain

Route #	First purification Method	Second purification Method	pH	NaCl	Catalytic activity
"1"	Ni-NTA	-	8.0	250mM	moderate
"2"	Ni-NTA	cation-exchange	5.5	100-500mM	low
"3"	Ni-NTA	cation-exchange	8.0	0 mM	moderate
"4"	Ni-NTA	size-exclusion	5.5	137 mM	low
"5"	Ni-NTA	size-exclusion	7.4	137 mM	high
"6"	Ni-NTA	size-exclusion	8.0	137 mM	high
"7"	Ni-NTA	size-exclusion	5.5	0 mM	extremely low
"8"	Ni-NTA	size-exclusion	8.0	0 mM	low

Table S2. Kinetic values for H34 and other catalytic antibody light chains

Catalytic antibody		kcat (min^{-1})	Km (M)	kcat/Km ($min^{-1}M^{-1}$)	substrate
#7TR (route "5" (pH7.4)		2.5×10^{-3}	1.05×10^{-4}	2.38 x 10	Arg-pNA
L12 light chain		1.6×10^{-3}	5.3×10^{-5}	3.0×10	Pro-Phe-Arg-MCA[16)]
H34	Purification (I); Ni-NTA at pH8.0	5.5×10^{-2}	3.2×10^{-6}	1.7×10^{4}	FRET-PD-1
	Purification (II); cation exchange at pH5.5	5.8×10^{-3}	7.9×10^{-6}	7.4×10^{2}	FRET-PD-1
	Purification (III); size-exclusion at pH7.4	1.8×10^{-1}	3.1×10^{-6}	5.9×10^{4}	FRET-PD-1
aIgV		3.0×10^{-1}	8.0×10^{-5}	3.8×10^{3}	Aβ[34)]
